# Rethinking Knee Arthroplasty: Bicompartmental Unicompartmental Knee Arthroplasty as a Biomechanically Personalized, Function‐Preserving Solution for Knee Osteoarthritis

**DOI:** 10.1111/os.70406

**Published:** 2026-08-19

**Authors:** Yishu Liu, Yong Zhou, Xi Zhang, Shengyuan Yu, Bing Xue, Xuefeng Liu

**Affiliations:** ^1^ The Fourth Affiliated Hospital of Harbin Medical University Harbin Heilongjiang Province China

## Abstract

**Aims:**

Although total knee arthroplasty (TKA) remains the standard treatment for advanced knee osteoarthritis (OA), it often compromises joint proprioception and natural kinematics. Bicompartmental unicompartmental knee arthroplasty (bi‐UKA), which preserves both the cruciate ligaments and patellofemoral joint, offers a joint‐preserving alternative for selected patients with bicompartmental OA. This study aimed to compare the early‐ and mid‐term outcomes of bi‐UKA and TKA.

**Methods:**

This single‐center, prospective, real‐world observational study included patients with knee osteoarthritis who were treated between January and October 2022. Eligible patients underwent either bi‐UKA or TKA according to routine clinical decision‐making and patient preference. Functional outcomes were assessed using the Knee Society Score (KSS), Western Ontario and McMaster Universities Osteoarthritis Index (WOMAC), range of motion (ROM), timed up and go test (TUG), and 2‐min walk test over a 2‐year follow‐up period. Preference‐related selection bias was reduced using inverse probability of treatment weighting (IPTW) based on a five‐item preoperative preference assessment tool, and longitudinal outcomes were analyzed using IPTW‐weighted mixed‐effects models.

**Results:**

Eighty patients were included in this study (bi‐UKA: *n* = 35, TKA: *n* = 45). After inverse probability of treatment weighting (IPTW) adjustment, the bi‐UKA group showed more favorable early outcomes: higher 1‐month KSS functional scores (*p* = 0.001); better WOMAC scores at 1, 6, and 12 months (*p* < 0.05); greater ROM throughout follow‐up (*p* < 0.0001); shorter TUG times; and longer 2MWT distances at early time points. The Forgotten Joint Score (FJS) scores at 1 and 2 years also favored bi‐UKA, indicating greater satisfaction.

**Conclusion:**

Bi‐UKA was associated with more favorable early functional recovery. By preserving the key anatomical structures and enabling more physiological motion, bi‐UKA may be a feasible alternative in selected patients.

## Graphical abstract

A comparative study of bicompartmental unicompartmental knee arthroplasty (bi‐UKA) and total knee arthroplasty (TKA) in patients with bicompartmental osteoarthritis. The image illustrates key anatomical differences in implant design, outlines the study population (bi‐UKA: *n* = 35, TKA: *n* = 45), and highlights functional evaluation metrics including the Knee Society Score (KSS), Western Ontario and McMaster Universities Osteoarthritis Index (WOMAC), range of motion (ROM), timed up and go (TUG) test, and 2‐min walk test (2MWT). Results showed that bi‐UKA demonstrated significantly better early‐ and mid‐term outcomes in terms of mobility, pain relief, and patient satisfaction. The Knee Procedure Willingness Assessment Scale (KPWAS) was used to quantify preoperative surgical preferences. Overall, bi‐UKA preserves native anatomy and enables superior recovery in selected patients.

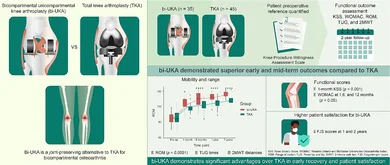

## 1 Introduction

Knee osteoarthritis (KOA) is a common degenerative disease among middle‐aged and elderly individuals, with an increasing incidence that significantly affects patient quality of life [1]. Total knee arthroplasty (TKA), considered the ‘gold standard’ for treating moderate‐to‐severe KOA, has shown excellent results in terms of pain relief. However, concerns remain regarding postoperative functional recovery, restoration of physiological motion patterns, and patient satisfaction. Recently, with the growing emphasis on knee‐preserving strategies, unicompartmental knee arthroplasty (UKA) has gained increasing attention because of its advantages of minimizing tissue trauma and facilitating faster recovery [2, 3]. The postoperative proprioception and motor coordination after UKA are similar to those in the natural knee.

Several studies have highlighted that the medial and lateral knee compartments are anatomically and kinematically independent. Osteoarthritic changes often occur asymmetrically across compartments [4, 5]. Some patients present with medial and lateral compartment involvement, whereas the patellofemoral joint remains relatively unaffected [6, 7]. Although TKA can relieve pain and improve function in such cases, extensive resection of the ligamentous structures and bone stock is required. In Asian cultures, deep knee flexion positions are more frequently required owing to cultural or religious practices [8].

Bicompartmental UKA (bi‐UKA) is a novel surgical technique. Bi‐UKA replaces both the medial and lateral compartments while preserving the core knee ligaments to restore function more precisely, adhere to knee‐preserving principles, and offer a more natural experience of postoperative motion. Bi‐UKA is generally considered for carefully selected patients with symptomatic osteoarthritis involving both the medial and lateral tibiofemoral compartments, while the patellofemoral joint remains relatively preserved and the anterior and posterior cruciate ligaments (PCLs) remain functionally intact. Its application also requires adequate bone stock, ligamentous stability, and the absence of severe fixed deformity or inflammatory joint disease. Therefore, careful preoperative imaging assessment and intraoperative confirmation of compartmental cartilage damage and ligament integrity are essential for appropriate patient selection.

However, in real‐world clinical decision‐making, systematic comparisons between bi‐UKA and TKA in terms of functional recovery and patient‐reported outcomes remain scarce. Therefore, this study aimed to introduce the Knee Procedure Willingness Assessment Scale (KPWAS) and utilize inverse probability of treatment weighting (IPTW) based on real‐world patient data to compare functional outcomes between bi‐UKA and TKA, further exploring the feasibility and advantages of bi‐UKA in appropriately selected patient populations. In addition to statistical significance, this study considered minimal clinically important differences (MCID) to determine whether observed effects were meaningful to patients [9]. Through this real‐world comparison, we sought to assess the clinical feasibility and potential functional value of bi‐UKA as an anatomy‐ and function‐preserving alternative to TKA and to provide preliminary evidence for the further development of knee‐preserving arthroplasty strategies.

## 2 Materials and Methods

This was a single‐center, real‐world, observational study. The study protocol was reviewed and approved by the Medical Ethics Committee of the Fourth Affiliated Hospital of Harbin Medical University (IRB approval No. 2021‐Ethics Review‐106). Patients who were treated at our outpatient clinic between January and October 2022 were consecutively screened. Eligible patients received bi‐UKA or TKA according to routine clinical decision‐making and patient preference rather than investigator‐assigned allocation. Longitudinal clinical outcomes were collected during postoperative follow‐up and analyzed using IPTW‐based methods to reduce preference‐related selection bias. As treatment decisions were made within routine clinical practice and shared decision‐making, no active intervention in patient treatment preference was performed by the investigators.

### 2.1 Patient Selection

#### 2.1.1 Inclusion Criteria

Patients aged 50–70 years diagnosed with KOA and treated in our department, irrespective of sex, were included. The indication for bi‐UKA in this study was osteoarthritic involvement of both the medial and lateral tibiofemoral compartments, with intact anterior and PCLs. Preoperative weight‐bearing radiographs and magnetic resonance imaging were used to evaluate the distribution and severity of osteoarthritis and to assess cruciate ligament integrity. Following joint exposure, suitability for bi‐UKA was further confirmed by direct inspection of cartilage degeneration in both the medial and lateral compartments and assessment of the morphology and functional integrity of the anterior cruciate ligament (ACL). Eligible patients exhibited medial and lateral compartmental KOA (Kellgren–Lawrence [KL] grade 2 or 3) without severe varus or valgus deformity (< 15°) and with intact ACL and PCL. All patients underwent standardized preoperative assessments and postoperative follow‐ups, including clinical evaluations, imaging studies, and complication tracking. Preoperative magnetic resonance imaging confirmed ligament integrity, and full‐length standing radiographs were used to assess lower limb alignment. The representative images are shown in Figure S1. Informed consent was obtained from all the participants in accordance with the study protocol.

#### 2.1.2 Exclusion Criteria

Patients with cruciate ligament injuries, rheumatoid arthritis, post‐traumatic arthritis, a history of osteotomy or bone disease, or severe comorbidities were excluded. Patients lacking follow‐up data or who experienced unrelated postoperative complications were also excluded.

All surgeries were performed by the same orthopedic surgeon, and all patients received standardized postoperative care and rehabilitation protocols.

### 2.2 Surgical Technique

#### 2.2.1 Bicompartmental Unicompartmental Knee Arthroplasty

We used the cemented LDK XU unicompartmental prosthesis system. Figure 1 illustrates the surgical approach and osteotomy instrumentation used. Based on the preoperative assessment and shared decision‐making process, patients assigned to the bi‐UKA group had already been considered suitable candidates for bicompartmental unicompartmental arthroplasty. Final intraoperative confirmation was performed after joint exposure. In our procedure, the lateral compartment was addressed first because this approach provided clearer exposure of the lateral articular surface and reduced obstruction by the patella, which can interfere with lateral compartment preparation when approached from the medial side.

**FIGURE 1:**
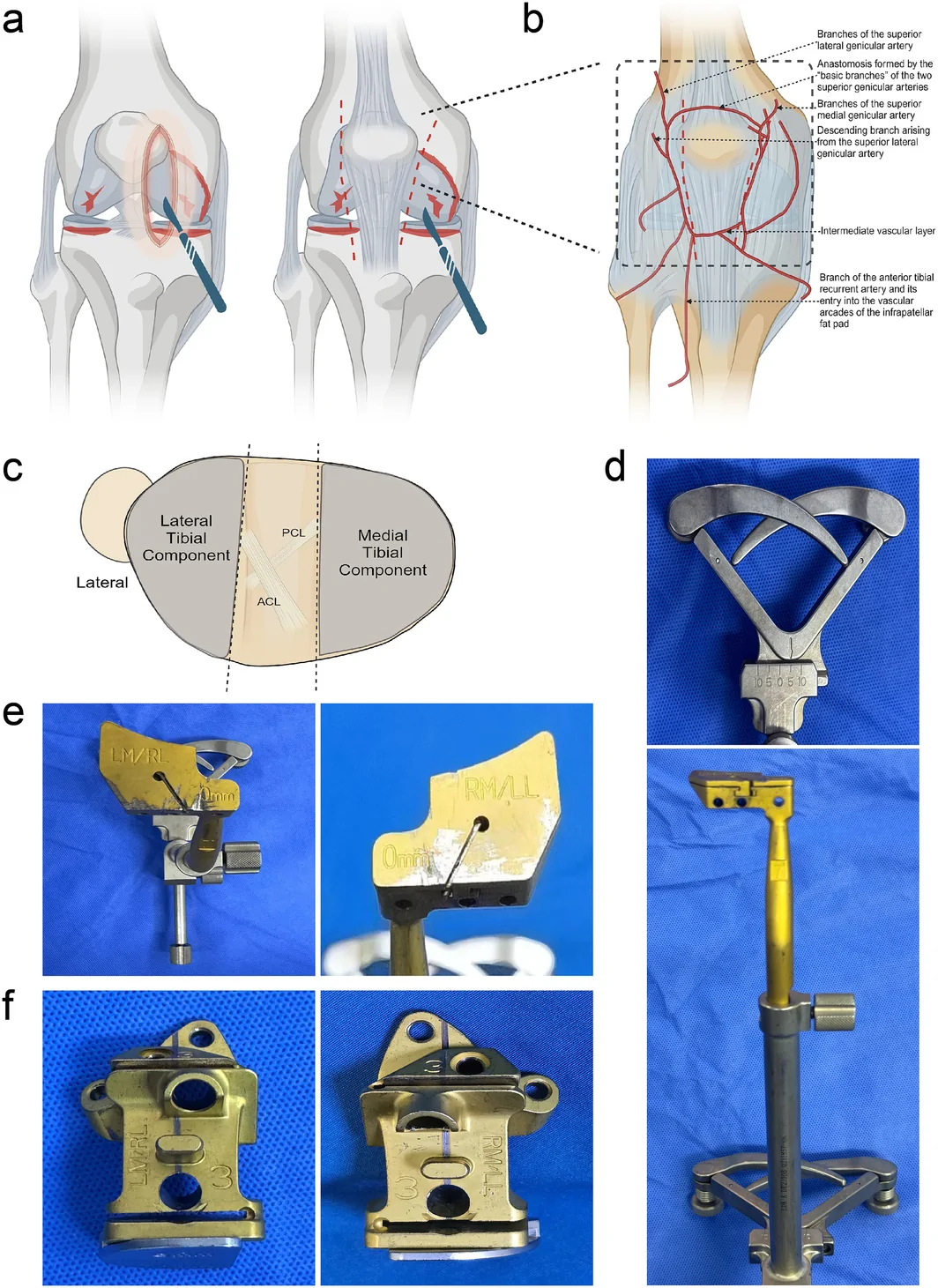
Illustration of surgical approach and instrumentation for bicompartmental unicompartmental knee arthroplasty (bi‐UKA). (a) Intraoperative approach and incision. A midline skin incision slightly medial to the patellar tendon was made, approximately 9 cm in length. The skin and subcutaneous tissues were incised sequentially down to the level of the patellar tendon. Medial and lateral arthrotomies were performed along both sides of the patellar tendon to access the medial and lateral compartments for bi‐UKA. (b) Vascular anatomy surrounding the patella. Illustrated are the arterial arcades formed by branches from the superior medial and superior lateral genicular arteries, which supply the peripatellar region. Particular care should be taken to preserve the branches of the superior medial genicular artery to maintain perfusion to the superomedial patella. Additionally, branches of the anterior recurrent tibial artery within the infrapatellar fat pad contribute to the deep vascular supply of the patellar tendon. Preservation of the fat pad and related ligamentous structures is recommended to maintain optimal postoperative vascular integrity. (c) Tibial resection direction and implant orientation. To better match the native tibial anatomy, the medial tibial component is implanted in slight external rotation, whereas the lateral component is positioned in slight internal rotation. This configuration facilitates the restoration of physiological femorotibial kinematics during flexion–extension. (d) Adjustment of the distal fixation assembly. Due to obstruction by the patellar tendon anteriorly, lateral tibial resection requires lateral displacement of the ankle clamp to avoid impingement. This ensures anatomical alignment of the extramedullary guide and maintains horizontal resection of the tibial plateau. (e) Selection of tibial cutting guides. The left panel depicts the guide for the right lateral tibial plateau (right lateral, RL), whereas the right panel shows the guide for the right medial tibial plateau (right medial, RM). (f) Femoral condyle cutting guides. The left panel shows the femoral guide for the right lateral condyle (RL), and the right panel illustrates the guide for the right medial condyle (RM). The metallic spacer beneath each guide assists in balancing the flexion and extension gaps during femoral bone preparation.

A skin incision of approximately 9 cm was made, followed by layered dissection to expose the lateral compartment (Figure 1a). After lateral capsulotomy, the patella was gently displaced medially with a bone lever to allow inspection of the medial compartment. Bi‐UKA was confirmed when degenerative changes were identified in both the medial and lateral tibiofemoral compartments and when the anterior and PCLs were macroscopically intact in morphology and tension, consistent with bicompartmental KOA.

The blood supply to the patellar tendon arises from the peripatellar arterial network, including branches from the superior medial genicular artery, the anastomotic arc formed by the superior genicular arteries, superior lateral genicular artery branches, descending branches from the same artery, intermediate vascular plexus, and anterior tibial recurrent artery branches that contribute to the infrapatellar fat pad arcade [10]. In conventional TKA or unicompartmental arthroplasty, only one side of the peripatellar soft‐tissue envelope is typically disturbed. In contrast, bi‐UKA requires arthrotomy and operative manipulation on both sides of the patellar tendon. Therefore, preservation of the patellar tendon blood supply was considered particularly important in this technique. Special attention was given to preserving the medial blood supply by minimizing retraction and maintaining the integrity of the vastus medialis insertion. The joint capsule was placed below the muscle margin to protect the descending genicular artery (Figure 1b). Deep dissection near the medial patellar tendon was avoided to reduce the risk of ischaemia. We also preserved the infrapatellar fat pad and ACL‐related synovial structures as much as possible to maintain the peripatellar vascular environment.

The medial and lateral compartments were prepared sequentially. The medial tibial baseplate was externally rotated, and the lateral baseplate was internally rotated to align with the anatomical orientations (Figure 1c). Figures 1d,e illustrate the adjustments required for the tibial cutting guides because of patellar tendon obstruction during lateral platform preparation. The distal femoral cutting guide for each compartment was selected accordingly (Figure 1f). For tibial resection, the posterior slope was set at approximately 7° on the medial side and 10° on the lateral side.

We adopted a suspended leg position with 20° hip flexion and 20° external rotation for natural knee flexion, allowing dynamic assessment of flexion‐extension gap balance and compartmental stability throughout the range of motion (ROM). Medial and lateral unicompartmental procedures were then performed sequentially. Medial preparation was performed with standard bone resection to restore the native joint line and achieve balanced flexion and extension gaps without excessive soft‐tissue release. Medial UKA was completed using standard techniques, including osteophyte removal, tibial and femoral osteotomies (Figures 2d,e), bone preparation, trial implantation, and ligament balancing (Figure 2f). For the lateral compartment, because of the distinct kinematics of the lateral compartment and the interference caused by the patellar tendon, particular care was taken in guide positioning, rotational alignment, and trial reduction of the lateral side. Lateral arthrotomy was extended from the patellar margin to Gerdy's tubercle (Figures 2g,h). Osteophytes were resected (Figures 3a,b), the tibial platform was cut with the intended posterior slope, and the femoral preparation was completed according to standard unicompartmental principles (Figures 3c–e). Trial components were inserted sequentially, and flexion‐extension gap balance, compartmental tension, coronal stability, patellar tracking, and overall limb alignment were reassessed before definitive implantation.

**FIGURE 2:**
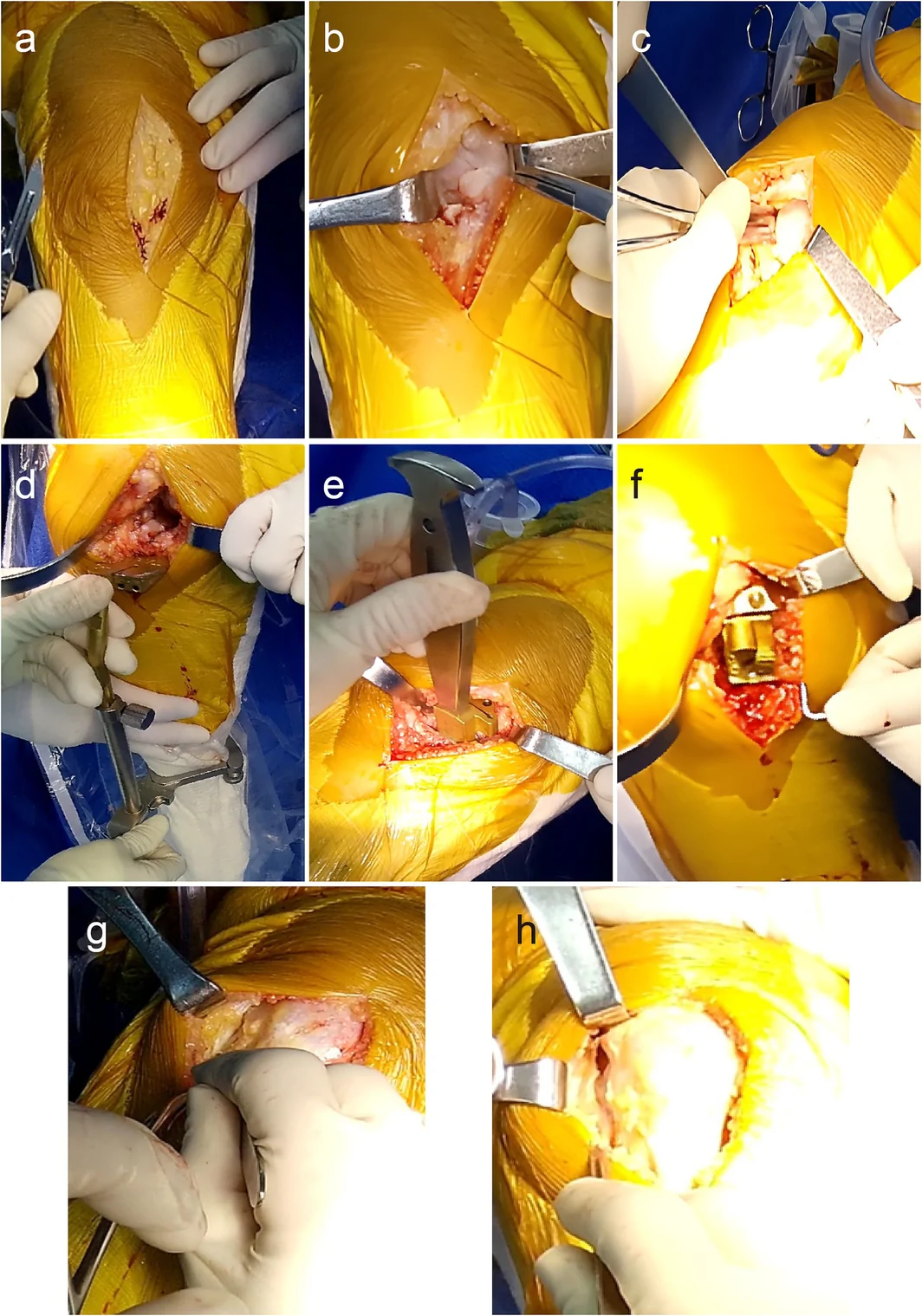
Intraoperative steps of bicompartmental unicompartmental knee arthroplasty (bi‐UKA). (a) A 9‐cm midline skin incision slightly medial to the patella is made. The skin and subcutaneous tissue are dissected layer by layer down to the level of the patellar tendon. (b) Medial parapatellar arthrotomy is performed along the medial edge of the patellar tendon, exposing the medial compartment, with visible osteophyte formation and cartilage degeneration. (c) Intraoperative exploration of both medial and lateral compartments and the anterior and posterior cruciate ligaments confirms consistency with preoperative assessments and eligibility for bi‐UKA. (d) Placement of the medial tibial cutting guide and execution of a precise tibial plateau resection on the medial side. (e) Distal femoral cutting guide is positioned to adjust the extension gap, followed by distal femoral resection to ensure proper prosthetic alignment in full extension. (f) Placement of the medial femoral condyle cutting guide and completion of resection of the medial condyle. (g) Dissection is performed along the lateral patellar bursa to release soft tissues and facilitate exposure of the lateral patellar tendon. (h) A lateral parapatellar arthrotomy is performed, successfully exposing the lateral compartment with full visualization of the lateral cartilage surface and osteophytes, providing adequate access for lateral unicompartmental implantation.

**FIGURE 3:**
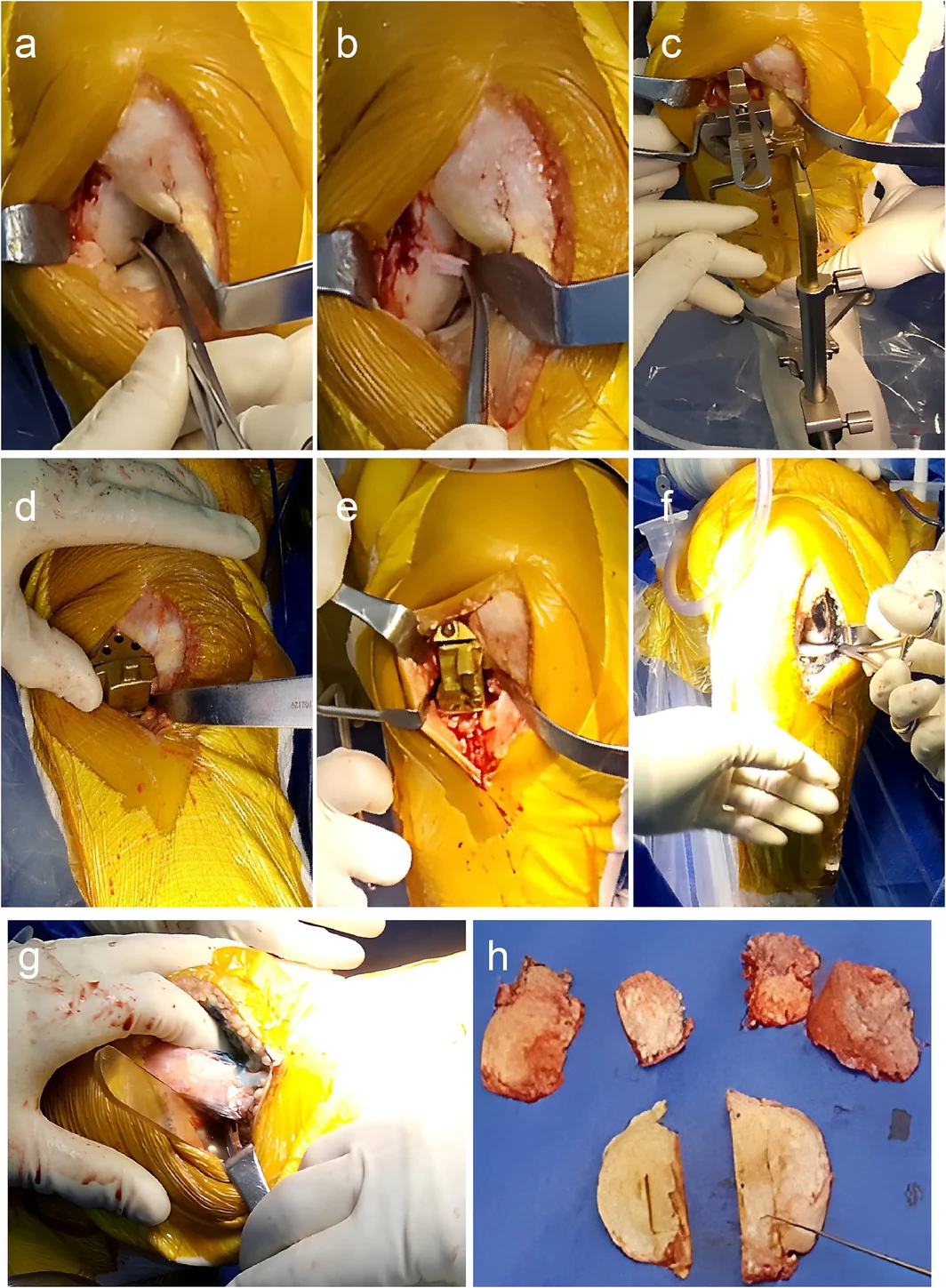
Intraoperative steps and bone resection overview in bicompartmental unicompartmental knee arthroplasty (bi‐UKA). (a) Intraoperative evaluation of the lateral compartment to assess cartilage degeneration and osteophyte formation, determining concordance with preoperative surgical indications. (b) Notable cartilage defects are observed in the lateral compartment, consistent with preoperative imaging and clinical assessments. (c) Placement of the lateral tibial cutting guide, followed by directional adjustment and resection of the lateral tibial plateau. (d) Positioning of the distal femoral cutting guide for the lateral condyle; resection is completed following proper adjustment of the extension gap. (e) The lateral femoral condyle cutting guide is positioned, and resection of the condyle is performed. (f) Trial implantation and final fixation of the medial unicompartmental prosthesis are performed, ensuring intraoperative stability and range of motion. (g) Implantation of the lateral unicompartmental prosthesis is completed, followed by a comprehensive assessment of bilateral compartment mobility, alignment, and prosthesis congruency. (h) Intraoperative visualization of resected bone segments demonstrates minimal bone removal, underscoring the bone‐preserving nature of bi‐UKA and its potential benefits for postoperative functional recovery.

After irrigation, a multimodal periarticular injection (100 mg ropivacaine, 5 mg dexamethasone, 0.3 mL epinephrine, 40 mg methylene blue in saline to 60 mL total volume) was administered. Implantation followed the sequence: medial tibia → medial femur → lateral femur → lateral tibia (Figures 3f,g). All components were fixed using bone cement according to the standard cementing principles routinely applied in knee arthroplasty. The tourniquet was released before closure to assess patellar tendon perfusion. Layered closure was then performed, and the wound was dressed using elastic compression bandages. Figure 3h shows the amount of bone resected.

#### 2.2.2 TKA Surgical Technique

We used the MicroPort Evolution CS Total Knee System for all TKA procedures. A standard midline incision of approximately 18 cm in length was made, followed by layered dissection through the skin and subcutaneous tissues to the joint capsule. The capsule was incised medially along the patella and everted to expose the joint. The ACL was sacrificed as required by the prosthesis design.

An intramedullary rod was inserted along the femoral axis and connected to the distal femoral cutting guide to perform distal femoral resection. A tibial cutting guide was then positioned with a posterior slope of 7°, and proximal tibial resection was completed. The extension gap was then measured. Femoral component sizing was performed, and a 4‐in‐1 femoral cutting block was used for additional femoral resections. Trial femoral and tibial components along with appropriate spacers were placed to ensure balanced extension and flexion gaps and proper alignment. The rotational angle of the tibial platform was confirmed, and a keel slot was prepared.

After thorough irrigation, a periarticular injection (‘cocktail’) was administered around the joint capsule, composed of ropivacaine 100 mg, dexamethasone 5 mg, epinephrine 0.3 mL, methylene blue 40 mg, and normal saline to a total volume of 60 mL. The definitive tibial baseplate, insert, and femoral components were implanted sequentially, and fixation was ensured after cement curing.

### 2.3 Follow‐Up and Data Collection

Demographic variables included age, sex, and body mass index (BMI). Perioperative parameters, such as incision length, operative time, and hospital stay duration, were collected. Hb levels were measured preoperatively and again on postoperative day 2, and the postoperative Hb drop was calculated as the difference between these two values as a surrogate indicator of perioperative blood loss. Postoperative assessments were conducted at 1, 6, 12, and 24 months using the Knee Society Score (KSS) and Western Ontario and McMaster Universities Osteoarthritis Index (WOMAC) for functional evaluation. The KSS evaluates clinical knee status and functional ability, with higher scores indicating better function, whereas the WOMAC assesses pain, stiffness, and physical function in osteoarthritis, with lower scores indicating better outcomes.

Functional mobility and gait performance were evaluated using the 2‐min walk test (2MWT) and timed up and go (TUG) test. In these assessments, longer walking distances in the 2MWT and shorter times in the TUG test indicate better mobility and physical performance. Pain and patient‐reported satisfaction were measured using the visual analogue scale (VAS) and Forgotten Joint Score (FJS). VAS scores were recorded on postoperative days 1 and 3 to reflect early postoperative pain and surgical trauma, with lower VAS values representing less pain. The FJS scores were assessed at 12 and 24 months postoperatively to evaluate long‐term joint awareness and subjective satisfaction and to minimize early‐stage bias, where higher scores indicate greater satisfaction and less joint awareness. All data were collected by the clinical team as part of a standardized follow‐up protocol.

To distinguish statistically significant differences from those of clinical relevance, we adopted established MCID thresholds for each outcome measure based on published literature. A ≥ 15 point improvement in the WOMAC total score was considered clinically meaningful [11]. For the KSS, a ≥ 9 point increase in the Knee Score and a ≥ 10 point increase in the Function Score represented the MCID [12]. A ≥ 5° gain in ROM [13], a ≥ 2.27 s reduction in TUG test time, and a ≥ 14.96 m increase in 2MWT [14] were considered the thresholds for functional improvement. For the FJS [15], a ≥ 13.7 point increase was used to reflect a meaningful reduction in joint awareness. These MCID criteria were used to assist in interpreting the magnitude and clinical impact of observed differences between groups.

### 2.4 Design and Application of the Knee Procedure Willingness Assessment Scale

In this real‐world observational setting, procedure allocation was preference‐related rather than investigator‐assigned. Therefore, a five‐item preference assessment tool (KPWAS) was used to quantify preoperative procedural preference and support propensity score weighting in this real‐world observational analysis. This scale comprises five dimensions that influence patient preferences during preoperative decision‐making:

1. *Importance of Postoperative Mobility:* Level of concern for returning to deep flexion tasks (e.g., squatting, kneeling, climbing stairs), scored 0 (not important) to 4 (very important).
2. *Desire to Preserve Native Knee Structures:* Degree of preference for retaining natural knee anatomy, scored 0 (not desired) to 4 (highly desired)
3. *Sensitivity to Surgical Risks:* Concerns about surgical complications, such as infection or prosthesis loosening, scored 0 (not concerned) to 4 (very concerned)
4. *Proactive Information Seeking:* Whether the patient actively researched bi‐UKA or TKA through the Internet, literature, or peers scored 0 (no) or 1 (yes).
5. *Decision‐Making Authority:* Whether the surgical decision was primarily made by the physician, shared, or by the patient scored 0 (physician‐led), 1 (shared), or 2 (patient‐led).

#### 2.4.1 Application Protocol

The scale was administered during the preoperative consultations. Composite scores reflected the patients' preoperative procedural preferences and served as behavioral and psychological variables in subsequent statistical adjustments.

#### 2.4.2 Reliability and Validity Testing

Preliminary evaluation suggested acceptable internal consistency and content validity; detailed psychometric results are provided in the Supporting Information.

### 2.5 Propensity Score Weighting Analysis

IPTW was used to adjust for potential confounders owing to nonrandomized group allocation. A logistic regression model was constructed using the five KPWAS items as covariates to estimate the probability $e_{i}$ of undergoing bi‐UKA (bi‐UKA, 1; TKA, 0). Individual $\mathrm{IPT}W_{i}$ values were calculated as $\frac{1}{e_{i}}$ (bi‐UKA group) or $\frac{1}{1-e_{i}}$ (TKA group), and the adjusted outcome values $Y_{\mathrm{ij}}$ at each time point *j* were derived as $Y_{\mathrm{ij}\left(\mathrm{adj}\right)}$:

$$Y_{\mathrm{ij}\left(\mathrm{adj}\right)}=Y_{\mathrm{ij}}\times \mathrm{IPT}W_{i}$$

These adjusted outcomes were used for between‐group comparisons of the clinical endpoints. By applying these weights, a weighted pseudo‐population was constructed in which the baseline characteristics of the two groups were balanced, thereby minimizing the influence of treatment selection bias.

### 2.6 Statistical Analyses

The normality of continuous variables was assessed using the Shapiro–Wilk test. Continuous variables are presented as means ± standard deviations (SD), whereas non‐normally distributed continuous variables are presented as medians with interquartile ranges (IQR) and categorical variables as frequencies and percentages. Baseline demographic and perioperative variables were compared using analysis of variance for normally distributed continuous data, the Mann–Whitney U test for non‐normally distributed continuous data, and the chi‐squared test for categorical data. To account for preference‐related selection bias in this non‐randomized real‐world study, IPTW derived from the propensity score model based on the KPWAS items was applied. Covariate balance before and after IPTW was assessed using absolute standardized mean differences (SMDs). Longitudinal postoperative outcomes were analyzed using IPTW‐weighted linear mixed‐effects models, including fixed effects for group, timepoint, and the group × timepoint interaction, with participant included as a random intercept. Model assumptions were evaluated by graphical inspection of residual plots. Pairwise post hoc comparisons were adjusted using the Sidak method. Adjusted least‐squares means and 95% confidence intervals are reported. All analyses were performed using GraphPad Prism 9.0 (GraphPad Software, San Diego, CA, USA) and R version 4.5.0 (R Foundation for Statistical Computing, Vienna, Austria), and a two‐sided *p*‐value < 0.05 was considered statistically significant.

## 3 Results

### 3.1 Patient Characteristics

A total of 463 patients were screened between January and October 2022, of whom 252 were diagnosed with KOA. Of these, 86 met the eligibility criteria and consented to participate (35 underwent bi‐UKA; 51 underwent TKA). By December 31, 2024, 80 patients had complete follow‐up data: 35 patients undergoing bi‐UKA and 45 patients undergoing TKA (six were excluded for incomplete follow‐up). The overall study flow is shown in Figure S3. The baseline and perioperative data are summarized in Table 1.

**TABLE 1:** Baseline demographic characteristics of patients.

	Bi‐UKA (*n* = 35)	TKA (*n* = 45)	*p*
Sex (*n*, %)			
Male	8 (22.9)	17 (37.8)	0.15
Female	27 (77.1)	28 (62.2)	
Age (years)	66.60 ± 6.27	67.89 ± 6.18	0.37
BMI	27.36 ± 2.06	26.63 ± 3.26	0.23

*Note:* This table summarizes the baseline demographic characteristics of patients. No statistically significant differences were observed between the bi‐UKA and TKA groups in terms of sex, age, or BMI.

### 3.2 Role of the KPWAS in Propensity Score Adjustment

To account for preference‐related differences in treatment allocation, the KPWAS was used to estimate propensity scores and derive inverse probability of treatment weights. Preliminary evaluation suggested acceptable content validity and internal consistency, supporting its use as an adjustment tool in this analysis. In the propensity score model, items related to postoperative mobility, preservation of native anatomy, and perceived surgical risk contributed most to treatment allocation. The resulting weights were generally stable, indicating acceptable balance potential across groups. Detailed psychometric findings and full propensity score modeling results are presented in Tables S1 and S2. Balance diagnostics demonstrated consistent reductions in the absolute SMDs of Q1–Q4 after IPTW, with decreases ranging from approximately 24.5% to 33.7% (Table S3). These findings indicate that the KPWAS‐based propensity score model improved comparability between the two groups with respect to the major preference‐related factors influencing procedure selection.

### 3.3 Knee Function Evaluation

#### 3.3.1 Perioperative and Patient‐Reported Outcomes

No statistically significant differences were observed between the bi‐UKA and TKA groups in baseline demographic characteristics, including age, sex, and BMI (Table 1). The perioperative and patient‐reported outcomes are presented in Table 2. Compared with TKA, bi‐UKA was associated with a shorter hospital stay (9.31 ± 1.51 vs. 10.80 ± 2.59 days, *p* = 0.0022), a shorter incision (9.13 ± 0.60 vs. 17.89 ± 1.68 cm, *p* < 0.0001), and a smaller decrease in hemoglobin on postoperative day 2 (16.28 ± 4.71 vs. 24.40 ± 8.45 g/L, *p* < 0.0001). However, the operative time was longer in the bi‐UKA group than in the TKA group (62.69 ± 7.21 vs. 50.13 ± 5.11 min, *p* < 0.0001), reflecting the greater technical complexity of the procedure.

**TABLE 2:** Perioperative and patient‐reported outcomes.

	Bi‐UKA	TKA	*p*
VAS 1D	0.89 ± 0.52	1.00 ± 0.76	0.43
VAS 3D	2.63 ± 0.96	3.42 ± 1.22	0.0019**
Hospital stay (day)	9.31 ± 1.51	10.80 ± 2.59	0.0022**
Incision length (cm)	9.13 ± 0.60	17.89 ± 1.68	< 0.0001****
Operative time (min)	62.69 ± 7.21	50.13 ± 5.11	< 0.0001****
Hb drop on postoperative 2 days, g/L	16.28 ± 4.71	24.40 ± 8.45	< 0.0001****
FJS 1Y	71.01 ± 13.41	55.19 ± 14.36	< 0.0001****
FJS 2Y	78.69 ± 12.05	70.05 ± 13.65	0.004**

*Note:* This table summarizes the perioperative characteristics, early postoperative pain, and patient‐reported joint awareness in the bi‐UKA and TKA groups. The bi‐UKA group had a significantly shorter hospital stay than the TKA group (9.31 ± 1.51 vs. 10.80 ± 2.59 days, **p = 0.0022), a shorter incision length (9.13 ± 0.60 vs. 17.89 ± 1.68 cm, ****p < 0.0001), and a smaller decrease in hemoglobin on postoperative day 2 (16.28 ± 4.71 vs. 24.40 ± 8.45 g/L, ****p < 0.0001). However, the operative time was significantly longer in the bi‐UKA group than in the TKA group (62.69 ± 7.21 vs. 50.13 ± 5.11 min, ****p < 0.0001). There was no significant between‐group difference in VAS pain scores on postoperative day 1 (0.89 ± 0.52 vs. 1.00 ± 0.76, *p* = 0.43), whereas the bi‐UKA group had a significantly lower VAS score on postoperative day 3 (2.63 ± 0.96 vs. 3.42 ± 1.22, **p = 0.0019). The FJS values were significantly higher in the bi‐UKA group at both 1 year (71.01 ± 13.41 vs. 55.19 ± 14.36, ****p < 0.0001) and 2 years (78.69 ± 12.05 vs. 70.05 ± 13.65, **p = 0.004), indicating lower postoperative joint awareness.

There was no significant between‐group difference in VAS pain scores on postoperative day 1 (0.89 ± 0.52 vs. 1.00 ± 0.76, *p* = 0.43), whereas the bi‐UKA group had a significantly lower VAS score on postoperative day 3 (2.63 ± 0.96 vs. 3.42 ± 1.22, *p* = 0.0019). No patient in either group required perioperative blood transfusion or developed wound‐healing complications, and all patients commenced assisted ambulation on postoperative day 2 according to the standardized rehabilitation protocol. The bi‐UKA group also showed higher FJS values at 1 year (71.01 ± 13.41 vs. 55.19 ± 14.36, *p* < 0.0001) and 2 years (78.69 ± 12.05 vs. 70.05 ± 13.65, *p* = 0.004), indicating lower postoperative joint awareness.

#### 3.3.2 Postoperative Functional Outcomes

Postoperative knee function was comprehensively assessed using the KSS, WOMAC, 2MWT, and TUG test scores. All outcomes were adjusted using IPTW weighting. The corresponding statistical trends and group comparisons are visually illustrated in Figure 4.

**FIGURE 4:**
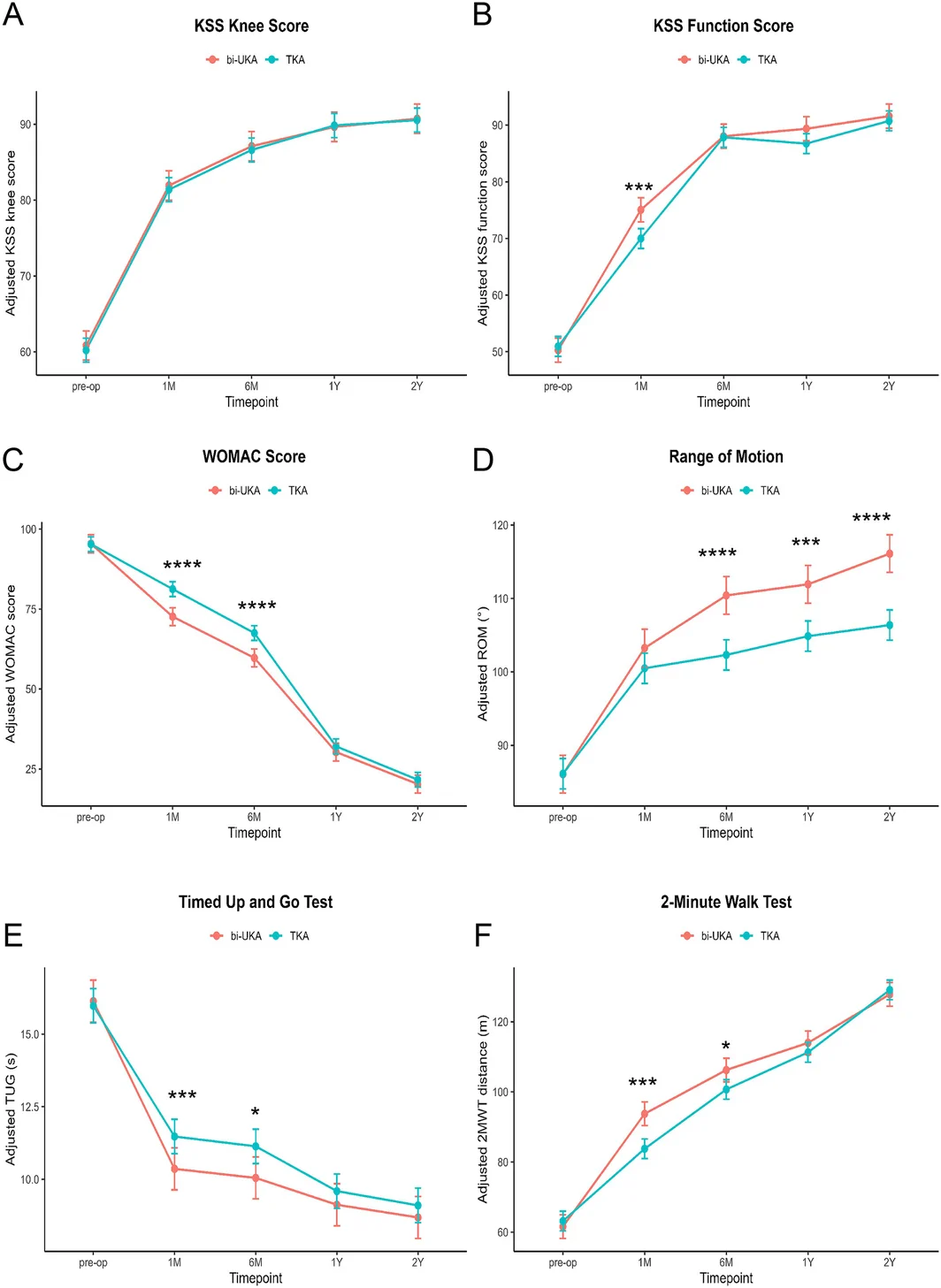
Comparison of postoperative knee function recovery between the bi‐UKA and TKA groups. This figure illustrates the longitudinal changes in multiple knee function parameters from baseline to 2 years postoperatively in the bi‐UKA and TKA groups, including the (a) KSS knee score, (b) KSS function score, (c) WOMAC score, (d) range of motion (ROM), (e) timed up and go (TUG) test, and (f) 2‐min walk test (2MWT). Box plots represent the distribution of values at each time point, with the median shown as the horizontal line and whiskers indicating the interquartile range. The line graphs depict the estimated marginal means over time. Asterisks indicate statistically significant differences between the two groups at specific time points (*p < 0.05, ***p < 0.001, ****p < 0.0001). The results demonstrated superior outcomes in the bi‐UKA group during the early postoperative period in terms of the KSS function, ROM, TUG, and WOMAC scores. Some advantages, particularly in ROM and 2MWT, persisted for 2 years postoperatively, suggesting the potential benefit of bi‐UKA in functional recovery and early resumption of daily activities.

The KSS scores, as shown in Tables 3 and 4, demonstrated progressive improvement in both groups throughout the follow‐up period. Notably, the bi‐UKA group exhibited superior early functional recovery at 1 month (*p* = 0.001), although group differences diminished over time (Figure 4a,b). The WOMAC scores in Table 5 showed statistically significant differences at 1 month, 6 months, and 1 year (*p* < 0.05), favoring the bi‐UKA group (Figure 4c). These differences remained significant even after the IPTW adjustment, supporting more favorable outcomes in the bi‐UKA group.

**TABLE 3:** Comparison of KSS knee joint scores.

KSSKNEE	Bi‐UKA	TKA	*p*	Bi‐UKA IPTW	TKA IPTW	*p*
Pre‐op	61.00 ± 6.65	59.69 ± 4.73	0.28	60.89 ± 6.89	60.36 ± 4.40	0.13
1 months	81.71 ± 5.48	81.35 ± 5.50	0.77	81.98 ± 5.48	81.53 ± 5.66	0.37
6 months	86.74 ± 6.26	86.02 ± 4.44	0.55	87.17 ± 6.13	86.76 ± 4.31	0.31
1 year	89.51 ± 4.72	89.47 ± 4.81	0.96	89.71 ± 4.69	89.99 ± 5.56	0.54
2 years	90.49 ± 5.42	91.07 ± 5.81	0.63	90.79 ± 5.47	90.69 ± 6.18	0.90

*Note:* This table presents the Knee Society Scores (KSS‐Knee) for the bi‐UKA and TKA groups at baseline and during the postoperative follow‐up, along with the results adjusted using inverse probability of treatment weighting (IPTW). Both groups demonstrated significant improvements from baseline at all postoperative time points. However, there were no statistically significant differences between the two groups at any time point before or after IPTW adjustment.

**TABLE 4:** Comparison of KSS knee joint function scores.

KSS function	Bi‐UKA	TKA	*p*	Bi‐UKA IPTW	TKA IPTW	*p*
Pre‐op	50.43 ± 6.25	49.22 ± 6.83	0.38	50.27 ± 6.43	50.94 ± 7.03	0.86
1 month	75.14 ± 6.27	70.44 ± 6.73	0.001***	75.06 ± 6.24	69.99 ± 7.38	0.03*
6 months	88.00 ± 5.24	86.78 ± 4.96	0.37	88.03 ± 5.15	87.85 ± 4.71	0.90
1 year	89.29 ± 6.98	88.22 ± 5.59	0.44	89.36 ± 6.95	86.74 ± 5.52	0.96
2 years	91.43 ± 5.56	91.44 ± 5.23	0.99	91.59 ± 5.42	90.75 ± 4.93	0.44

*Note:* This table summarizes the Knee Society Functional Scores (KSS‐Function) of the bi‐UKA and TKA groups at different time points. No significant difference was observed in the preoperative functional scores (*p* = 0.38). One month postoperatively, the bi‐UKA group demonstrated significantly higher scores than the TKA group (***p = 0.001), and this difference remained significant after IPTW adjustment (*p = 0.03). These findings suggest superior early functional recovery in the bi‐UKA group. By ≥ 6 months, the scores between the two groups converged, indicating no long‐term functional differences.

**TABLE 5:** WOMAC score comparison.

WOMAC	Bi‐UKA	TKA	*p*	Bi‐UKA IPTW	TKA IPTW	*p*
Pre‐op	95.29 ± 4.61	93.96 ± 4.74	0.45	95.40 ± 4.87	95.12 ± 5.15	0.33
1 month	73.14 ± 12.52	80.33 ± 3.61	0.0001****	72.61 ± 13.17	81.08 ± 4.01	0.0008****
6 months	60.09 ± 11.60	68.69 ± 5.08	< 0.0001****	59.73 ± 12.24	67.33 ± 5.43	0.0001****
1 year	29.97 ± 7.29	33.76 ± 9.24	0.03*	30.29 ± 7.21	31.92 ± 10.39	0.17
2 years	20.09 ± 6.02	21.67 ± 7.93	0.36	20.29 ± 6.06	21.49 ± 7.27	0.79

*Note:* This table presents the Western Ontario and McMaster Universities Osteoarthritis Index (WOMAC) scores used to evaluate pain, stiffness, and functional limitations in daily life for the bi‐UKA and TKA groups. No significant differences were observed preoperatively between the groups (*p* = 0.45). At 1 month, 6 months, and 1 year postoperatively, the bi‐UKA group showed significantly better WOMAC scores than the TKA group (*p* < 0.05) even after IPTW adjustment. By 2 years, the differences were no longer statistically significant, suggesting that the superior subjective recovery experience of bi‐UKA is most evident during the early‐to mid‐term postoperative period. (*p < 0.05, ****p < 0.0001).

Dynamic function tests further showed more favorable dynamic functional recovery in the bi‐UKA group, these tests are summarized in Tables S4, S5, and S6. The ROM was consistently higher in the bi‐UKA group at all postoperative time points than the TKA group (e.g., 6 M: 110.41° vs. 102.31°, *p* < 0.0001), and this remained significant after adjustment (*p* < 0.0001) (Figure 4d). The TUG test (Figure 4e) also demonstrated faster recovery of coordination and dynamic stability in the bi‐UKA group at 1 M and 6 M than in the TKA group (e.g., 1 M: 10.33 ± 1.29 s vs. 11.67 ± 2.4 s, *p* = 0.006). The 2MWT showed better walking ability in the bi‐UKA group than in the TKA group at 1 M, 6 M, and 1Y (e.g., 1 M: 93.49 ± 9.83 m vs. 85.42 ± 6.65 m, *p* = 0.001) (Figure 4f).

In summary, the bi‐UKA technique was associated with more favorable recovery outcomes than TKA in terms of early pain control, patient satisfaction, and both static and dynamic functional recoveries. In particular, assessments, such as the WOMAC, ROM, 2MWT, and TUG test, revealed the clinically meaningful benefits of bi‐UKA.

The surgical incision and early postoperative ROM are illustrated in Figure S2. The incision was approximately 9 cm in length, and the patient was able to achieve > 120° of knee flexion 1 month postoperatively, indicating satisfactory early functional recovery. Representative radiographic findings are shown in Figures 5 and 6. Preoperative anteroposterior and lateral radiographs demonstrated narrowing of both the medial and lateral joint spaces, osteophyte formation, and irregularity of the articular surfaces, consistent with bicompartmental osteoarthritis (Figures 5a and 6a). Immediate postoperative radiographs confirmed proper alignment and positioning of both the medial and lateral unicompartmental prostheses (Figures 5b and 6b). Follow‐up radiographs at 2 years postoperatively showed stable implant positioning with preserved joint space and no signs of loosening or periprosthetic osteolysis (Figures 5c and 6c), indicating favorable mid‐term outcomes.

**FIGURE 5:**
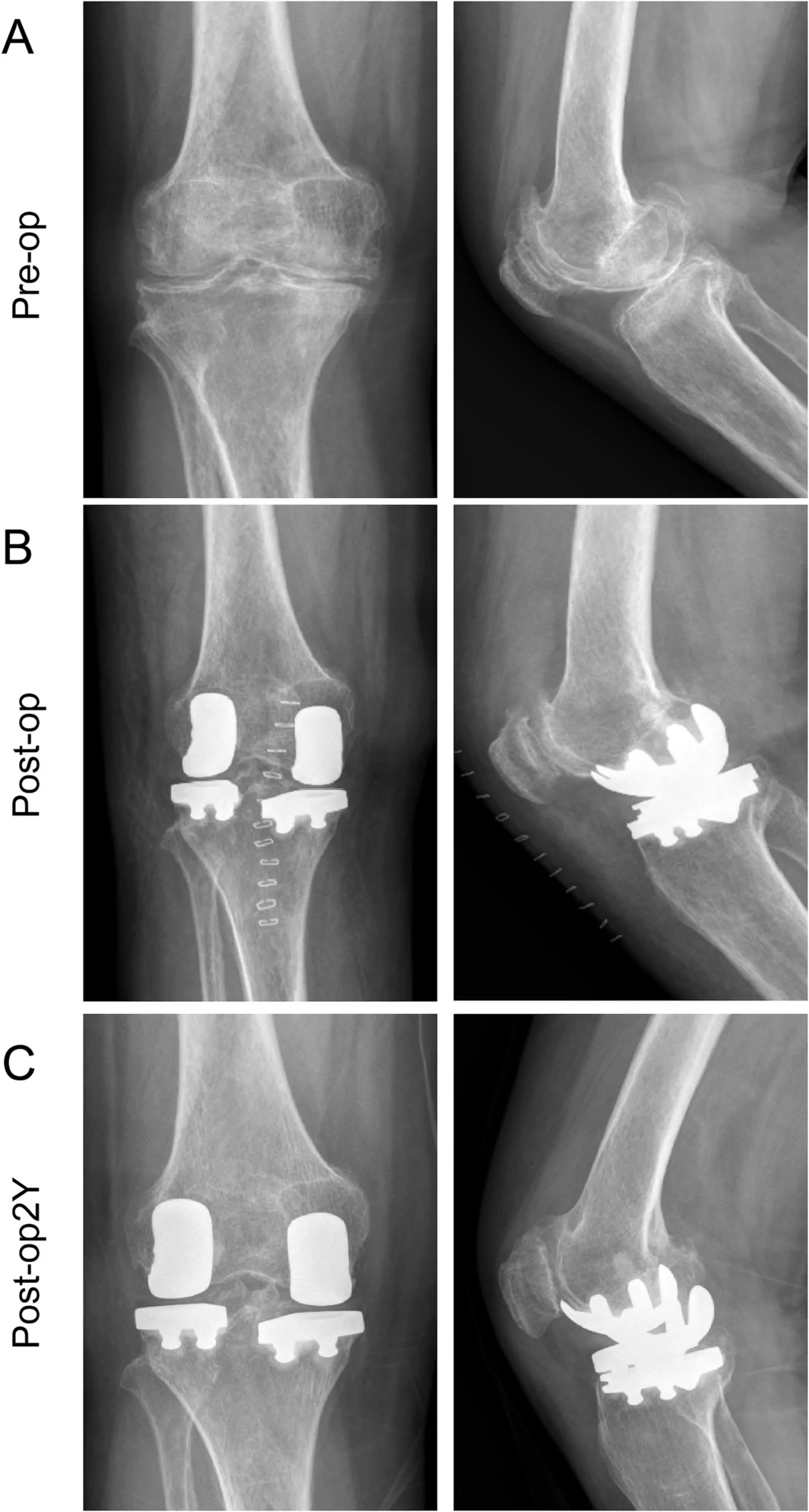
Radiographic comparison of the knee joint before and after bicompartmental unicompartmental knee arthroplasty (bi‐UKA). (a) Preoperative anteroposterior (AP) and lateral view radiographs of the knee show significant narrowing of both the medial and lateral joint spaces, irregularity of the articular surfaces, and osteophyte formation, indicative of bicompartmental osteoarthritic changes. (b) Immediate postoperative AP and lateral view radiographs demonstrate proper implantation of the medial and lateral unicompartmental prostheses, with good alignment and congruency between the components and joint surfaces. (c) AP and lateral view radiographs at 2 years postoperatively reveal stable implant positioning, preserved joint space, and no signs of loosening, migration, or periprosthetic complications. The bony margins show no evidence of osteolysis or bone resorption, indicating a favorable mid‐term radiographic outcome.

**FIGURE 6:**
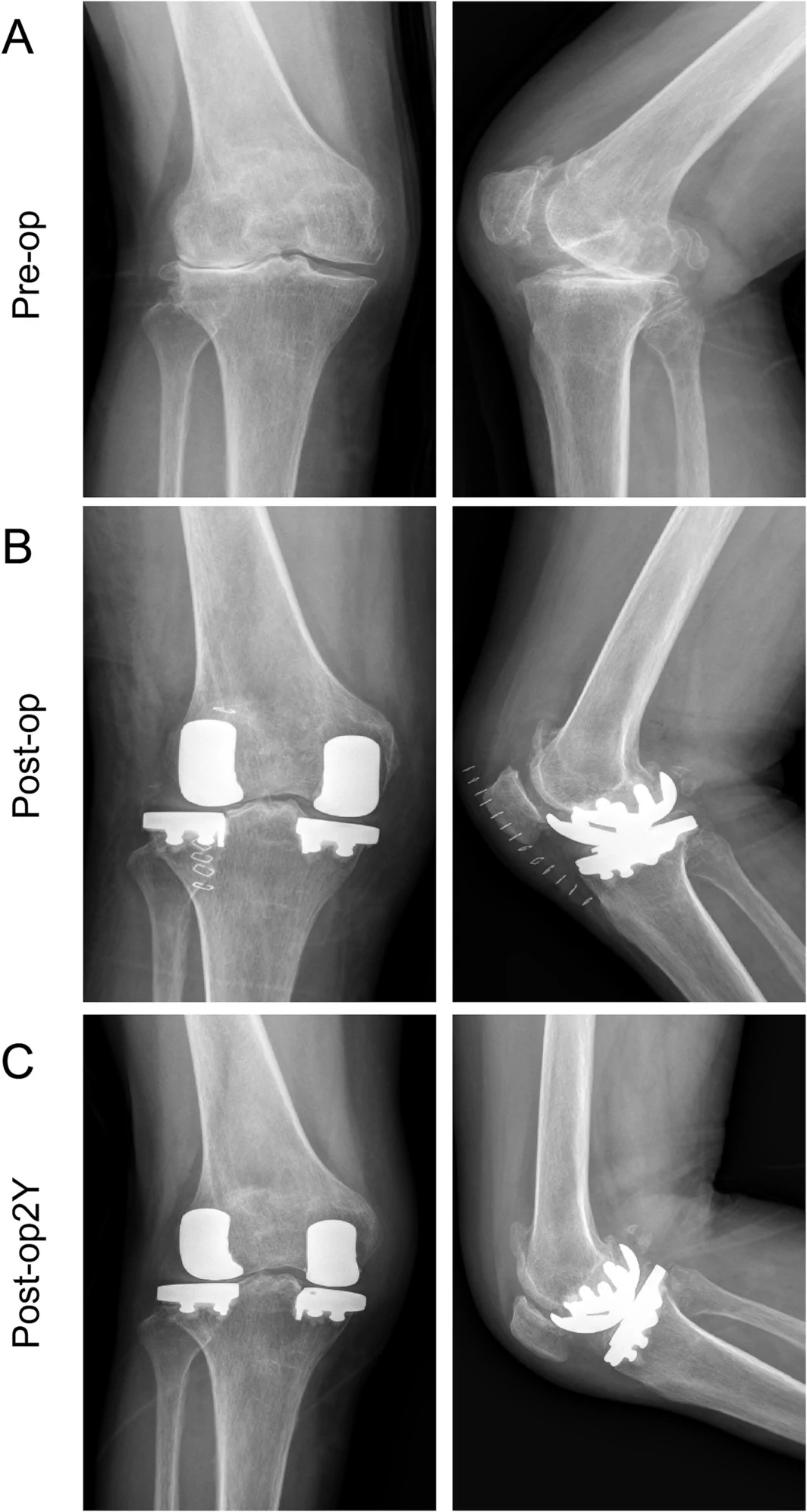
Representative radiographic follow‐up of another patient undergoing bicompartmental unicompartmental knee arthroplasty (bi‐UKA). (a) Preoperative anteroposterior (AP) and lateral radiographs demonstrate marked joint‐space narrowing in both the medial and lateral tibiofemoral compartments, accompanied by osteophyte formation and irregularity of the articular surfaces, consistent with bicompartmental knee osteoarthritis. (b) Immediate postoperative AP and lateral radiographs show satisfactory positioning and alignment of the medial and lateral unicompartmental components, with appropriate component congruency. (c) AP and lateral radiographs obtained 2 years postoperatively demonstrate maintained component position and alignment, without obvious radiographic evidence of prosthetic loosening, migration, periprosthetic fracture, or osteolysis, indicating a favorable mid‐term radiographic outcome.

## 4 Discussion

### 4.1 Early Functional Multidimensional Recovery Advantages After Bi‐UKA

This study compared the postoperative functional outcomes between bi‐UKA and TKA. By incorporating propensity score‐based IPTW and mixed‐effects models, we aimed to reduce measured baseline selection bias and improve between‐group comparability. Our findings suggest that bi‐UKA was associated with more favorable postoperative functional recovery across multiple domains. These differences were particularly evident during the early postoperative period, highlighting the potential short‐term benefits of bi‐UKA. Among these measures, the WOMAC appeared to be more sensitive, likely owing to its multidimensional assessment of pain, stiffness, and physical function. Therefore, the WOMAC may better reflect patient‐perceived recovery than more clinician‐oriented measures such as the KSS. The favorable patterns observed in ROM, TUG, and 2MWT further suggest potential functional benefits of bi‐UKA. Although some between‐group differences diminished during later follow‐up, these findings are consistent with the potential value of preserving native knee anatomy and promoting more physiological postoperative function. Although ROM remained statistically greater in the bi‐UKA group, the clinical relevance of the absolute differences should be interpreted cautiously. The between‐group difference at 1 month did not exceed the prespecified 5° reference threshold, whereas the differences at 6 months, 1 year, and 2 years were approximately 7°–9°. However, because this threshold was derived primarily from within‐patient change rather than direct between‐procedure comparisons, these findings should not be regarded as definitive evidence of clinically meaningful superiority.

### 4.2 From Prosthetic Design to Compartment‐Preserving Knee Reconstruction

An underlying reason for the superior functional performance of bi‐UKA may be due to its anatomical fidelity, biomechanical congruence, and preservation of soft tissue structures. Anatomical and kinematic studies have established that the medial and lateral compartments of the knee operate with distinct motion patterns and should not be considered a single kinematic unit. During flexion, the lateral femoral condyle exhibits greater posterior rollback than the medial side, contributing to asymmetric loading and wear patterns; anteromedial degeneration is typical in the medial compartment, whereas posterolateral wear predominates in lateral osteoarthritis [4, 5, 16] Notably, computed tomography‐based studies have reported significant inter‐compartmental differences in native tibial slope. For instance, in one study of 2395 knees, 364 patients undergoing lateral UKA had an average preoperative lateral slope of 8.0° ± 3.3°, with 28.3% exceeding 10° [17]. However, standard TKA tibial cuts often apply a uniform 7° posterior slope bilaterally, which fails to replicate individualized anatomy in most patients. One anatomical study reported that only approximately 15% of TKA cases achieved matching slopes on the medial and lateral sides postoperatively [18]. Finite element modeling confirmed that altering the lateral slope had a greater impact on femorotibial kinematics than altering the medial slope [19, 20, 21]. In response, we adopted a differential slope approach in bi‐UKA: 7° on the medial and 10° on the lateral tibial plateau, to recreate native rollback patterns and minimize deviation from physiological motion.

Compared with posterior‐stabilized or cruciate‐retaining TKA, the CS‐type TKA system attempts to replicate native medial pivot mechanics via a ball‐and‐socket design. Although studies have reported improved midflexion stability and patient satisfaction with CS designs [22], standard resection techniques and loss of the PCL can lead to kinematic conflict, limited deep flexion, and unnatural joint sensation. In contrast, bi‐UKA retains both the ACL and PCL, allows for compartment‐specific reconstruction, and avoids the forced symmetry imposed by monolithic TKA implants. Bi‐UKA also overcomes the challenges in balancing the flexion–extension gaps inherent in TKA. This mismatch can lead to limited flexion and discomfort [23], and may partly explain why up to 20% of patients undergoing TKA remain dissatisfied postoperatively—often due to overcorrection of natural varus/valgus alignment or anterior instability from ACL resection [24]. Moreover, the ability of bi‐UKA to preserve the native cruciate ligaments facilitates proprioception, neuromuscular control, and joint stability, contributing to faster rehabilitation and improved gait. This aligns with the emerging biomimetic and kinematic alignment (KA) [25] concepts in modern knee arthroplasty. KA‐based approaches demonstrate better early functioning and fewer adaptation‐related complaints. These issues may not be addressed by current TKA designs but can be mitigated by the individualized and tissue‐sparing features of bi‐UKA. The surgical technique for bi‐UKA requires precise compartmental evaluation, individualized planning, and technical expertise. As such, a certain learning curve is expected, particularly in achieving accurate implant positioning and joint line balance. In this study, all procedures were performed by a senior orthopedic surgeon experienced in UKA, thereby minimizing variability related to operator skill.

Collectively, the advantages of bi‐UKA are underpinned by three core mechanisms: [1] preservation of key anatomical structures, maintenance of proprioception, and kinematic loops; [2] individualized osteotomy based on KA principles that better replicate physiological joint trajectories; and [3] a minimally invasive surgical approach that limits soft tissue trauma and accelerates postoperative recovery.

### 4.3 Future Development and Clinical Validation of Bi‐UKA

Although the present findings support the potential clinical value of bi‐UKA, further validation is required before its broader application. A key strength and design innovation of the present study was its preference‐sensitive, real‐world comparison of bi‐UKA and TKA in patients with bicompartmental KOA. This was a real‐world observational study in which treatment selection reflected routine clinical practice, shared decision‐making, and patient preference rather than investigator‐directed allocation. By preserving the actual clinical decision‐making process, this design enabled bi‐UKA to be evaluated not merely as an isolated surgical technique, but as a compartment‐preserving alternative to TKA within the clinical pathway in which treatment decisions are made. Furthermore, the combination of this clinically authentic treatment‐selection framework with IPTW and longitudinal mixed‐effects modeling allowed the study to retain its real‐world relevance while analytically addressing the inherent non‐randomness of treatment allocation. Although IPTW was used to reduce measured preference‐related differences, potentially relevant factors such as baseline activity level, patient expectations, socioeconomic characteristics, and adherence to rehabilitation were not included in the model. These unmeasured factors may have influenced both treatment selection and postoperative recovery; therefore, residual confounding cannot be excluded, and the results should not be interpreted as definitive causal effects.

The relatively recent adoption of bi‐UKA at our institution resulted in a limited sample size and a relatively short follow‐up period. In addition, the single‐center and single‐surgeon design, although ensuring consistency in patient selection, surgical technique, and postoperative management, may limit the generalizability of the findings. The observed outcomes may also partly reflect the experience of the operating surgeon and the specific LDK XU unicompartmental prosthesis and MicroPort Evolution total knee system used in this study. Furthermore, bi‐UKA remains unsuitable for patients with severe deformity, advanced bone loss, or KL grade 4 disease, and direct comparison with bicruciate‐retaining TKA was not performed because of its limited regional use. Future multicenter studies involving larger cohorts, different surgeons and implant systems, and longer follow‐up are required to assess the reproducibility and long‐term durability of bi‐UKA. Postoperative three‐dimensional imaging and gait analysis may further clarify the effects of compartment‐specific slope reconstruction on prosthetic mechanics and knee function.

## 5 Conclusion

In this real‐world observational cohort, bi‐UKA was associated with more favorable early functional recovery and patient‐reported outcomes than TKA in appropriately selected patients with bicompartmental KOA. These findings provide preliminary real‐world evidence supporting the feasibility of bi‐UKA, but larger prospective studies with longer follow‐up are required before firmer comparative conclusions can be drawn.

## Author Contributions

**Yong Zhou:** methodology. **Yishu Liu:** conceptualization, writing – original draft, visualization, software, project administration, data curation. **Xuefeng Liu:** funding acquisition, writing – review and editing, supervision, resources. **Shengyuan Yu:** methodology, investigation. **Xi Zhang:** methodology, investigation. **Bing Xue:** methodology, visualization, formal analysis, validation.

## Funding

The authors have nothing to report.

## Ethics Statement

This study was approved by the Ethics Committee of the Fourth Affiliated Hospital of Harbin Medical University (Approval No. 2021‐伦理审查–106). All procedures were conducted in accordance with the ethical standards of the institution.

## Conflicts of Interest

The authors declare no conflicts of interest.

## Supporting information


**Figure S1:** Preoperative MRI and full‐length radiograph for surgical planning and patient inclusion. (a, b) Sagittal MRI views showing intact anterior and posterior cruciate ligaments. (c) Coronal MRI indicating degeneration in both medial and lateral compartments. (d) Preoperative full‐length standing AP view radiograph demonstrating acceptable lower limb alignment. These imaging findings were consistent with the inclusion criteria for bi‐compartmental unicompartmental knee arthroplasty.
**Figure S2:** Postoperative incision appearance and active knee flexion at 1 month. (a) Postoperative photographs showing the surgical incision, with a typical length of approximately 9 cm. (b) At 1 month postoperatively, the patient achieved active knee flexion exceeding 120°, indicating favorable early functional recovery and preserved joint mobility.
**Figure S3:** Flow diagram of patient.
**Table S1:** IPTW and Predicted Probabilities for Each Patient Based on Propensity Score Model.
**Table S2:** Logistic regression analysis of propensity score assessment items.
**Table S3:** Balance diagnostics of the five KPWAS items before and after IPTW.
**Table S4:** Comparison of ROM.
**Table S5:** 2MWT comparison.
**Table S6:** TUG test comparison.

## Data Availability

Data can be obtained from the corresponding author for reasonable reason.
