# Supplementary material for: Rethinking Knee Arthroplasty: Bicompartmental Unicompartmental Knee Arthroplasty as a Biomechanically Personalized, Function‐Preserving Solution for Knee Osteoarthritis
Source: Orthop Surg. 2026 Aug 19;18(10):2101–17. doi: 10.1111/os.70406 (PMC13488483; doi:10.1111/os.70406)
Supplement: Supplementary file 1 [file OS-18-2101-s001.docx]

**Figure S1.** **Preoperative MRI and full-length radiograph for surgical planning and patient inclusion.** (a, b) Sagittal MRI views showing intact anterior and posterior cruciate ligaments. (c) Coronal MRI indicating degeneration in both medial and lateral compartments. (d) Preoperative full-length standing AP view radiograph demonstrating acceptable lower limb alignment. These imaging findings were consistent with the inclusion criteria for bi-compartmental unicompartmental knee arthroplasty.

**Figure S2. Postoperative incision appearance and active knee flexion at 1 month.** (a) Postoperative photographs showing the surgical incision, with a typical length of approximately 9 cm. (b) At 1 month postoperatively, the patient achieved active knee flexion exceeding 120°, indicating favorable early functional recovery and preserved joint mobility.

**Figure S3. Flow diagram of patient.**

Patients with knee osteoarthritis treated between January and October 2022 were screened for eligibility. Eligible patients who provided informed consent underwent either bi-UKA or TKA according to routine clinical assessment and shared decision-making. Clinical assessments were performed preoperatively and at 1, 6, 12, and 24 months postoperatively. After exclusion of patients with incomplete follow-up data, 35 patients in the bi-UKA group and 45 patients in the TKA group were included in the final IPTW-weighted longitudinal analysis. IPTW, inverse probability of treatment weighting.

**Table S1. IPTW and Predicted Probabilities for Each Patient Based on Propensity Score Model**

| Outcome | Predicted probability | IPTW |
| --- | --- | --- |
| 1 | 0.942214 | 1.06133 |
| 1 | 0.587685 | 1.701591 |
| 1 | 0.999999 | 1.000001 |
| 1 | 0.645509 | 1.549166 |
| 1 | 0.680502 | 1.469504 |
| 1 | 0.680502 | 1.469504 |
| 1 | 0.680502 | 1.469504 |
| 1 | 0.992582 | 1.007473 |
| 1 | 0.937281 | 1.066916 |
| 1 | 0.999998 | 1.000002 |
| 1 | 0.999998 | 1.000002 |
| 1 | 1 | 1 |
| 1 | 0.998543 | 1.001459 |
| 1 | 0.998295 | 1.001708 |
| 1 | 0.998295 | 1.001708 |
| 1 | 0.972574 | 1.0282 |
| 1 | 0.999998 | 1.000002 |
| 1 | 0.54926 | 1.820632 |
| 1 | 0.54926 | 1.820632 |
| 1 | 0.997825 | 1.00218 |
| 1 | 0.916047 | 1.091647 |
| 1 | 0.855449 | 1.168977 |
| 1 | 0.999941 | 1.000059 |
| 1 | 0.83497 | 1.197648 |
| 1 | 0.999992 | 1.000008 |
| 1 | 0.999993 | 1.000007 |
| 1 | 0.996861 | 1.003149 |
| 1 | 0.9997 | 1.0003 |
| 1 | 0.754731 | 1.324975 |
| 1 | 0.518667 | 1.92802 |
| 1 | 0.999993 | 1.000007 |
| 1 | 0.999993 | 1.000007 |
| 1 | 0.964978 | 1.036293 |
| 1 | 0.993024 | 1.007025 |
| 1 | 0.797041 | 1.254641 |
| 0 | 0.078858 | 1.085609 |
| 0 | 0.000631 | 1.000632 |
| 0 | 2.84E-06 | 1.000003 |
| 0 | 1.99E-05 | 1.00002 |
| 0 | 4.24E-06 | 1.000004 |
| 0 | 0.001067 | 1.001068 |
| 0 | 0.000423 | 1.000423 |
| 0 | 0.001361 | 1.001363 |
| 0 | 1.7E-05 | 1.000017 |
| 0 | 0.003772 | 1.003786 |
| 0 | 0.05883 | 1.062507 |
| 0 | 2.17E-05 | 1.000022 |
| 0 | 0.645509 | 2.820944 |
| 0 | 0.001738 | 1.001741 |
| 0 | 2.17E-05 | 1.000022 |
| 0 | 0.771992 | 4.385802 |
| 0 | 0.010324 | 1.010432 |
| 0 | 0.046692 | 1.048979 |
| 0 | 0.005626 | 1.005658 |
| 0 | 0.014086 | 1.014287 |
| 0 | 0.843223 | 6.378485 |
| 0 | 0.000105 | 1.000105 |
| 0 | 0.078858 | 1.085609 |
| 0 | 1.28E-05 | 1.000013 |
| 0 | 0.001738 | 1.001741 |
| 0 | 0.068199 | 1.073191 |
| 0 | 0.865897 | 7.456955 |
| 0 | 0.041513 | 1.043311 |
| 0 | 9.1E-13 | 1 |
| 0 | 9.1E-13 | 1 |
| 0 | 9.1E-13 | 1 |
| 0 | 0.587685 | 2.425331 |
| 0 | 0.000138 | 1.000138 |
| 0 | 1.36E-12 | 1 |
| 0 | 0.000495 | 1.000495 |
| 0 | 4.17E-14 | 1 |
| 0 | 1.57E-11 | 1 |
| 0 | 6.03E-16 | 1 |
| 0 | 7.65E-10 | 1 |
| 0 | 6.01E-14 | 1 |
| 0 | 5.68E-07 | 1.000001 |
| 0 | 0.028927 | 1.029789 |
| 0 | 8.84E-06 | 1.000009 |
| 0 | 2.05E-07 | 1 |
| 0 | 1.88E-06 | 1.000002 |

This table presents the predicted probability of undergoing bi-UKA for each patient, calculated from the propensity score model derived from five preoperative patient preference dimensions KPWAS. The corresponding IPTW is also listed, which was used to construct the weighted cohort for causal inference. Patients with an outcome value of “1” received bi-UKA, while “0” indicates TKA.

**Table S2. Logistic regression analysis of propensity score assessment items**

| Question item | Estimated value | P value |
| --- | --- | --- |
| Q1 | 3.21±1.17 | 0.0058 (**) |
| Q2 | 5.93±2.25 | 0.0085 (**) |
| Q3 | 2.19±1.12 | 0.0493 (*) |
| Q4 | 3.83±2.15 | 0.0757 (ns) |
| Q5 | –1.79±1.23 | 0.1462 (ns) |

This table presents the estimated coefficients and P-values for the five questionnaire items (Q1–Q5) included in the logistic regression model used to predict the likelihood of patients undergoing bicompartmental unicompartmental knee arthroplasty (bi-UKA). Q1 (P=0.0058), Q2 (P=0.0085), and Q3 (P=0.0493) demonstrated significant associations with surgical preference, suggesting that they are key contributors to the propensity score calculation. No significant associations were observed between Q4 and Q5 (P=0.0757 and P=0.1462, respectively). These findings indicate that the model was appropriately constructed to support the subsequent inverse probability of treatment weighting (IPTW) for confounding adjustment.

The internal consistency of the scale was evaluated using Cronbach’s α, yielding a coefficient of 0.748 in a pilot sample (N=30), indicating good reliability. Content validity was assessed via expert review (N=7 senior orthopaedic surgeons), with item-level content validity index (CVI) (I-CVI) >0.78 and scale-level CVI (S-CVI/Ave) =0.944. Expert agreement (Cr<0.25) confirmed this consensus.

**Table S3. Balance diagnostics of the five KPWAS items before and after IPTW**

| KPWAS item | SMD before IPTW | SMD after IPTW |
| --- | --- | --- |
| Q1 | 1.540 | 1.095 |
| Q2 | 1.760 | 1.328 |
| Q3 | 0.728 | 0.483 |
| Q4 | 0.591 | 0.433 |
| Q5 | 0.079 | 0.106 |

Standardized mean differences (SMDs) were calculated for the five KPWAS items before and after application of the IPTW weights derived from the propensity score model. Lower absolute SMD values indicate improved between-group comparability. After weighting, the absolute SMDs for Q1–Q4 were consistently reduced, demonstrating that KPWAS-based IPTW attenuated the preference-related differences between the bi-UKA and TKA groups. Q5 showed only a small between-group difference before weighting and remained close to the conventional balance threshold after weighting.

**Table S4. Comparison of ROM**

| ROM° | Bi-UKA | TKA | P value | Bi-UKA IPTW | TKA IPTW | P value |
| --- | --- | --- | --- | --- | --- | --- |
| Pre-op | 86.42±7.23 | 84.67±10.25 | 0.28 | 86.08±7.62 | 86.14±10.09 | 1 |
| 1M | 103.00±7.58 | 99.67±6.94 | 0.04(*) | 103.25±7.62 | 100.50±8.16 | 0.41 |
| 6M | 110.29±8.94 | 101.44±7.13 | <0.0001 (****) | 110.41±8.82 | 102.31±7.00 | <0.0001 (****) |
| 1Y | 111.89±5.72 | 104.44±6.44 | <0.0001 (****) | 111.93±5.58 | 104.87±5.69 | 0.0002 (***) |
| 2Y | 116.17±3.87 | 107.44±5.05 | <0.0001 (****) | 116.10±3.73 | 106.38±5.04 | <0.0001 (****) |

This table presents changes in knee range of motion (ROM) in the bi-UKA and TKA groups from baseline to 2 years postoperatively. No significant preoperative difference was observed either before or after IPTW adjustment (unadjusted P=0.28; IPTW-adjusted P>0.9999). Before weighting, the bi-UKA group showed greater ROM at all postoperative time points. After IPTW adjustment, the between-group difference was not statistically significant at 1 month (103.25±7.62° vs. 100.50±8.16°, P=0.41), but remained significant at 6 months (P<0.0001), 1 year (P=0.0002), and 2 years (P<0.0001). These findings suggest that bi-UKA was associated with greater ROM during the intermediate follow-up period, although the clinical relevance of the absolute differences should be interpreted cautiously.

**Table S5. 2MWT comparison**

| 2MWT | Bi-UKA | TKA | P value | Bi-UKA IPTW | TKA IPTW | P value |
| --- | --- | --- | --- | --- | --- | --- |
| Pre-op | 62.40±10.70 | 62.11±7.51 | 0.88 | 61.60±11.44 | 63.85±9.81 | 0.95 |
| 1M | 93.48±9.66 | 85.42±6.58 | 0.0002 (***) | 93.78±9.89 | 84.46±6.83 | 0.001 (***) |
| 6M | 105.83±8.71 | 101.33±7.42 | 0.03 (*) | 106.26±8.43 | 101.37±8.37 | 0.03 (*) |
| 1Y | 113.83±8.28 | 111.78±9.37 | 0.32 | 114.00±8.11 | 111.95±8.62 | 0.40 |
| 2Y | 127.57±11.15 | 126.27±10.33 | 0.52 | 127.84±11.27 | 129.74±13.30 | 0.21 |

This table summarises the results of the 2-min walk test (2MWT) used to assess walking capacity and lower limb endurance in the bi-UKA and TKA groups. No significant difference was observed preoperatively between the two groups (P=0.88). At 1 and 6 months postoperatively, the bi-UKA group showed significantly greater walking distances than the TKA group (P=0.0002, ***; P=0.03, *), with significance maintained after IPTW adjustment. These findings indicate that bi-UKA is associated with a more rapid and substantial improvement in walking ability, particularly in the early postoperative period.

**Table S6. TUG test comparison**

| TUG | Bi-UKA | TKA | P value | Bi-UKA IPTW | TKA IPTW | P value |
| --- | --- | --- | --- | --- | --- | --- |
| Pre-op | 16.28±4.57 | 15.54±3.09 | 0.33 | 16.13±4.45 | 16.08±3.09 | 0.43 |
| 1M | 10.33±1.27 | 11.67±2.37 | 0.006 (***) | 10.35±1.28 | 11.57±2.21 | 0.01 (**) |
| 6M | 10.01±1.25 | 11.04±1.26 | 0.03 (*) | 10.04±1.22 | 11.23±1.32 | 0.02 (*) |
| 1Y | 9.12±0.93 | 9.51±0.95 | 0.42 | 9.11±0.93 | 9.69±0.87 | 0.29 |
| 2Y | 8.71±1.25 | 9.00±1.12 | 0.55 | 8.68±1.27 | 9.20±1.04 | 0.50 |

This table presents the timed up and go (TUG) test outcomes, which evaluate postoperative dynamic stability and lower limb coordination by measuring the time required for patients to rise from a seated position, walk 3 m, turn around, and sit back down. No significant difference was observed between the groups preoperatively (P=0.33). At 1 and 6 months postoperatively, the bi-UKA group demonstrated significantly shorter TUG test times than the TKA group (P=0.006, ***; P=0.03, *), indicating a faster recovery of mobility and coordination. These results support the advantages of the bi-UKA approach for early rehabilitation.
